# Supplementary material for: Development of social behavior in young zebrafish
Source: Front Neural Circuits. 2015 Aug 18;9:39. doi: 10.3389/fncir.2015.00039 (PMC4539524; doi:10.3389/fncir.2015.00039)
Supplement: Supplementary file 6 [file DataSheet1.DOCX]

**Supplementary Figures**

**Supplementary Figure 1**

**Antisocial fish and behaviours in absence of social cues**

**A,** Table showing the age of the fish (one, two, three weeks) used for experiments and the corresponding range of body lengths used to select fish for the experiments. **B,** Example of tracking of a single three week old fish, in the absence (left) and presence (right) of multiple conspecifics (SC), showing a “non-social” aversive response to the SC. **C,** Schematic depicting body orientation of the observer test fish relative to the SC chamber. Polar histograms, averaged across all tested fish, of body orientations of the observer fish during the acclimation period. Thin lines indicate two standard errors from the mean (SEM) (one week: n=143, two weeks: n=151, three weeks: n=181). **D,** Histograms of SPIs during acclimation periods for the fish shown in Fig. 1e. **E,** Example of tracking of a three week old fish during two consecutive acclimation periods in the absence of a SC. **F,** Histogram of SPIs during two consecutive acclimation periods. The similar SPI values for Ac vs no SC during test period (Wilcoxon signed-rank test of paired samples p = 0.86) show that fish do not develop a bias for either chamber arm simply by spending more time in the behavioural chamber. Mean SPI within parentheses.

**Supplementary Figure 2**

**SPIs during acclimation periods for various experimental conditions.**

**A,** Histograms of SPIs of the fish shown in Fig. 2c during acclimation. **B,** Histogram of SPI during acclimation (left column) and SC (right column) periods in a chamber in which transparent windows were replaced with white opaque barriers. **C,** Histograms of SPIs of the fish shown in Fig. 2d during acclimation. **D,** Histograms of SPIs of the fish shown in Fig. 4 during acclimation. Mean SPI within parentheses.

**Movies**

**Movie 1**

Example of three week old fish simultaneously tracked online in a 6-chamber arena using the Bonsai software^32^. The movie recorded at 100 Hz is gradually sped up to aid presentation. Tracking traces for each single fish are superimposed on the movie and colour-coded offline. Only fish that were active during the acclimation phase and showed little or no bias for chamber arms were tested during the presentation of SC phase. Consequently fish such as the one top left which showed a strong preference for one arm of the chamber, or fish that didn’t swim very much during the first acclimation 15 minutes of imaging were not analysed.

**Movie 2**

Example movie of a one week old fish imaged at 100 Hz in the presence of multiple conspecifics (2X sped playback).

**Movie 3**

Example movie of a social three week old fish imaged at 100 Hz in the presence of multiple conspecifics.

**Movie 4**

Example movie of a non-social three week old fish imaged at 100 Hz in the presence of multiple conspecifics. Note how the tested fish seems to actively avoid the arm of the chamber with conspecifics.

**Movie 5**

Example movie of a three week old observer fish imaged at 100 Hz, but played 8X slower, in the presence of a SC fish. Note how the observer and the SC exhibit periods of movement synchrony.
