# Supplementary figures and images for: Development of social behavior in young zebrafish
Source: Front Neural Circuits. 2015 Aug 18;9:39. doi: 10.3389/fncir.2015.00039 (PMC4539524; doi:10.3389/fncir.2015.00039)

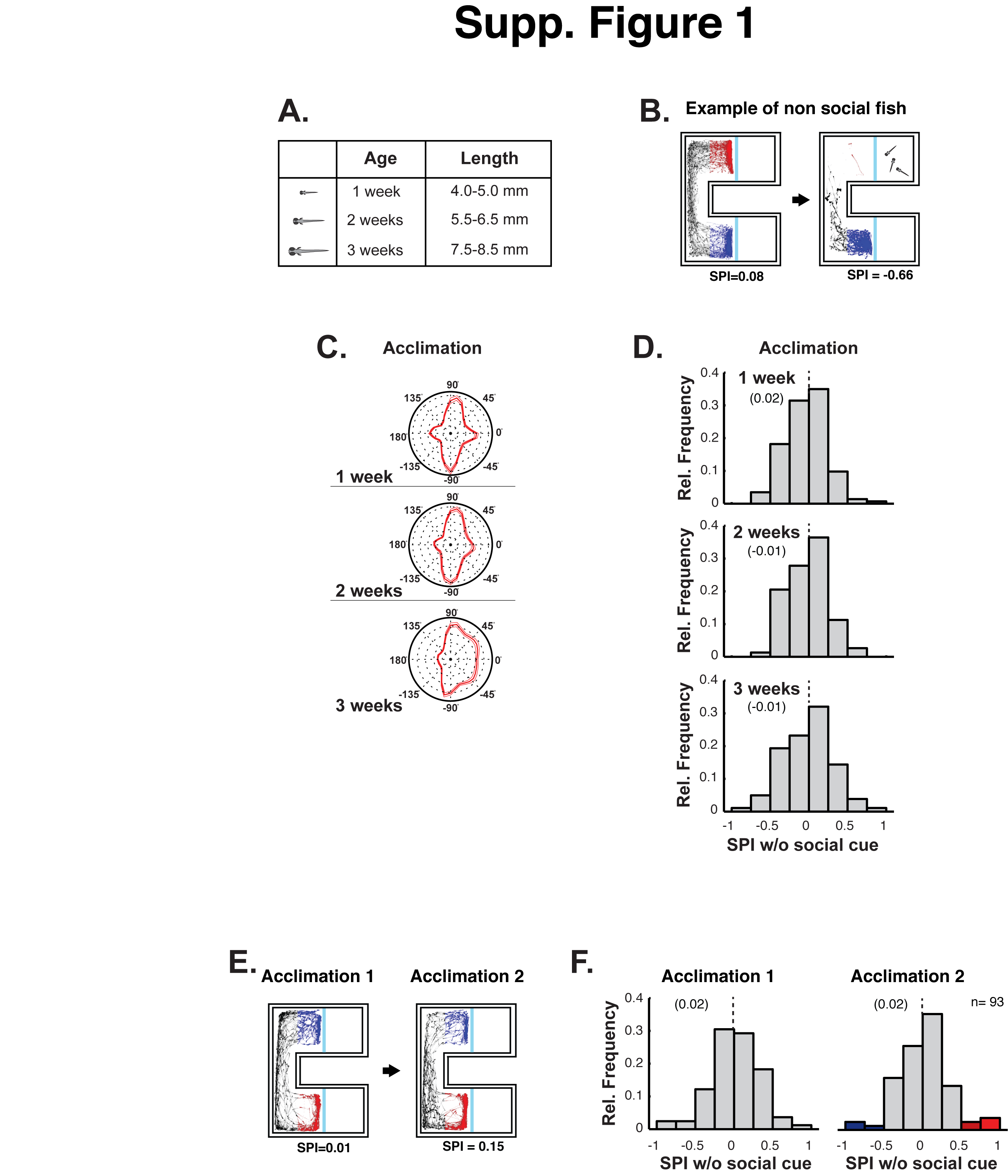

Supplement: Supplementary file 7 [file Image1.TIF]

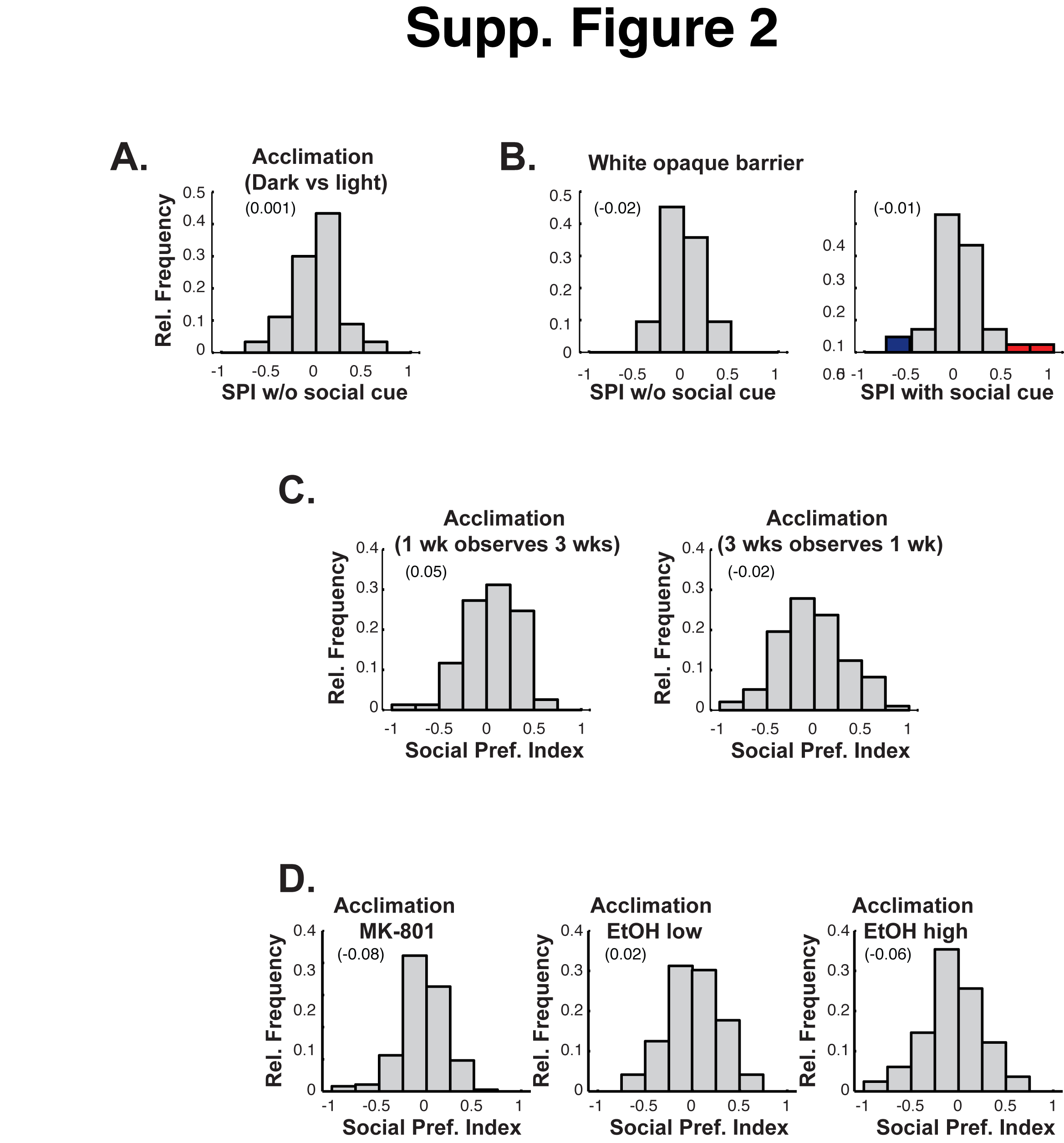

Supplement: Supplementary file 8 [file Image2.TIF]
